# Clinical symptoms and their relationship with cognitive impairment in elderly patients with depressive disorder

**DOI:** 10.3389/fpsyt.2022.1009653

**Published:** 2022-10-10

**Authors:** Zhenguo Wu, Guanli Su, Wenting Lu, Lin Liu, Zixuan Zhou, Bingchuan Xie

**Affiliations:** ^1^Department of Psychiatry, The First Hospital of Hebei Medical University, Shijiazhuang, China; ^2^Mental Health Institute of the Hebei Medical University, Shijiazhuang, China; ^3^Department of Neurology, The First Hospital of Hebei Medical University, Shijiazhuang, China

**Keywords:** geriatric depression disorder, cognitive impairment, correlation analysis, risk factors, cognitive

## Abstract

**Objective:**

To evaluate the correlation between clinical symptoms and cognitive impairment in elderly patients with depressive disorder.

**Methods:**

In this retrospective study, a total of 123 elderly patients with depressive disorder admitted to our hospital from January 2020 to February 2021 were included. Patients' cognitive function was assessed by the Montreal Cognitive Assessment Scale (MoCA). According to the combination of cognitive impairment or not, patients were divided into the combined group (64 cases) and the depressive disorder group (59 cases). In addition, 70 healthy people who came to our hospital for physical examination during the same period were randomly selected as the healthy group.

**Results:**

The incidence of severe cognitive impairment in the combined group (33, 51.56%) was significantly higher than that in the depression group (19, 32.20%), the difference was statistically significant (*P* = 0.003). The incidence of somatization symptoms, suicidal tendency, retardation of thinking, diminution of energy, anxiety and sleep disorder in the combined group were higher than that in the depressive disorder group with significant difference [30 (56.88%) vs. 16 (27.12%), *P* = 0.024; 12 (18.75%) vs. 3 (5.08%), *P* = 0.021; 33 (51.56%) vs. 14 (23.73%), *P* = 0.002; 37 (57.81%) vs. 23 (38.98%), *P* = 0.029; 42 (65.63) vs. 25 (42.37), *P* = 0.011; 50 (78.13) vs. 42 (71.19), *P* = 0.031, respectively]. Spearman rank correlation analysis suggested that somatic symptom, mood change, suicidal tendency, retardation of thinking, diminution of energy, anxiety, and sleep disorder were negatively correlated with cognitive impairment, respectively (*r* =-0.161, −0.672, −0.262, −0.871, −0.421, −0.571, −0.512, *P* < 0.001).

**Conclusion:**

The clinical symptoms of depressive disorder were negatively correlated with cognitive impairment. Somatic symptoms, suicidal tendency, retardation of thinking, diminution of energy, anxiety, and sleep disorder were the risk factors for cognitive impairment.

## Introduction

It was reported that China's aging population was about 18.1 percent at the end of 2019 and is expected to reach 28 percent by 2040 (1). With the increase of the elderly population, the psychological state of elderly patients has gradually received more attention (2). Geriatric depressive disorder refers to a mental disorder occurring after the age of 60 with persistent depressive mood as the main clinical phase, with a high incidence of 3.5–7.5% (3). The main characteristics of geriatric depressive disorder are high disability rate, high recurrence rate, and high suicide rate. It was reported that the disability rate was about 18%, the recurrence rate was about 80%, and the suicide rate was about 10–15%, as a result, geriatric depression disorder not only has a serious impact on the quality of life of patients but also increases the burden on patients and society (4). Previous studies have suggested that the incidence of depressive disorder accompanied by cognitive dysfunction is as high as 50–75%, if depressive disorder accompanied by cognitive dysfunction is temporary, it can be recovered with the relief of depressive mood, so it is also called pseudodementia (5).

The geriatric depressive disorder has a high prevalence, but low consultation rate, when developed into severe cognitive impairment, seriously affects the quality of life of the elderly and their spouses. The report suggested that more than 80 percent of older adults with depression in the United States seek care in primary care settings, often undiagnosed at the time of the visit (6). Therefore, it is of great clinical significance to timely distinguish elderly patients with depressive disorder and determine whether there is cognitive impairment for timely and effective symptomatic treatment and to avoid exacerbation of the condition (7). At present, there are few clinical studies on the clinical characteristics of elderly patients with depression disorder complicated with cognitive impairment. Based on this, this study aimed to evaluate the correlation between clinical symptoms and cognitive impairment and to explore the risk factors of cognitive impairment in elderly patients with depressive disorder.

## Materials and methods

### Study patients

This study retrospectively included 123 elderly patients with the depressive disorder who were admitted to our hospital from January 2020 to February 2021. The Montreal Cognitive Assessment Scale (MoCA) (8) was used to assess whether the patients with combined cognitive impairment were divided into the combined group, and the patients with single depressive disorder were divided into the depressive disorder group. Another seventy healthy people who were randomly selected to our hospital for physical examination at the same time were set as the healthy group for comparison.

#### Inclusion criteria

(1) The diagnosis of depressive disorder was in line with the diagnostic criteria for depression in the International Classification of Diseases (ICD-10) (9), the diagnosis was made by two psychiatrists with intermediate or higher professional titles; (2) Patients whose Geriatric Depression Scale (4) (GDS) score was ≥11; (3) No drugs such as escitalopram were used before admission; (4) Informed consent was signed by patients and their families.

#### Exclusion criteria

(1) Patients complicated with serious physical diseases, brain organic diseases, alcohol or drug dependence; (2) Patients complicated with mental retardation, Alzheimer's diseases, secondary depression, and bipolar disorder; (3) Patients complicated with heart, lung, kidney, and other organ diseases; (4) Depression caused by non-dependent substances; (5) Patients with systemic diseases and complications; (6) Patients with a history of alcohol or drug abuse or dependence within the past 2 years.

The study was approved by the Ethical Committee, and the informed consent forms were obtained from all patients.

### General information collection

Age, sex, marital status, education level, family history of depression, and causes of depression were collected for all subjects. Clinical symptoms of depressive disorder were collected, including somatic symptoms, mood change, suicidal tendency, retardation of thinking, diminution of energy, anxiety, and sleep disorder. Medication history and chronic disease history were collected. Chronic diseases to be screened included chronic bronchitis, chronic obstructive pulmonary disease, emphysema, cholelithiasis, cataract, chronic renal insufficiency, prostate disease, musculoskeletal disease, cerebrovascular disease, diabetes, and hyperlipidemia.

### Evaluation of depressive disorder

The severity of patients' depressive disorder was assessed by the GDS scale (4). The scale includes 30 subitems such as depressed mood, reduced activity, and irritability. Each item is given a score of 1 and the full score is 30. Depression is rated on three scales: 0–10 for normal, 11–20 for mild depression, and 21–30 for moderate to severe depression.

### Assessment of cognitive impairment

Cognitive impairment was assessed by the MoCA (8). The scale consists of 11 items with a total score of 30 points. A score <25 is classified as cognitive dysfunction; A score of ≥26 is considered normal. The patients were evaluated by 2 doctors by talking and observing, and each event was assessed for about 20 min. The physician didn't know the patient's disease information when evaluating the patient.

### Statistical analysis

Statistical analysis was performed using the SPSS software program (version 21.0; IBM Corp, Chicago, IL, USA). Normally distributed measurement data were expressed as mean ± standard deviation (SD). One-way ANOVA was used for comparison of measurement data between groups, and post-doc least significant difference (LSD) was used for comparison of data between and within groups. The enumeration data were expressed in frequency and percentage and were compared with the Chi-square test. Spearman rank correlation was used to analyze the relationship between clinical symptoms and cognitive impairment in patients with depressive disorder. Logistic regression was used to analyze the influencing factors of cognitive function in patients with depressive disorder. The test level α was 0.05 on both sides. *P* < 0.05 was considered statistically significant.

## Results

### General data

**Figure 1:**
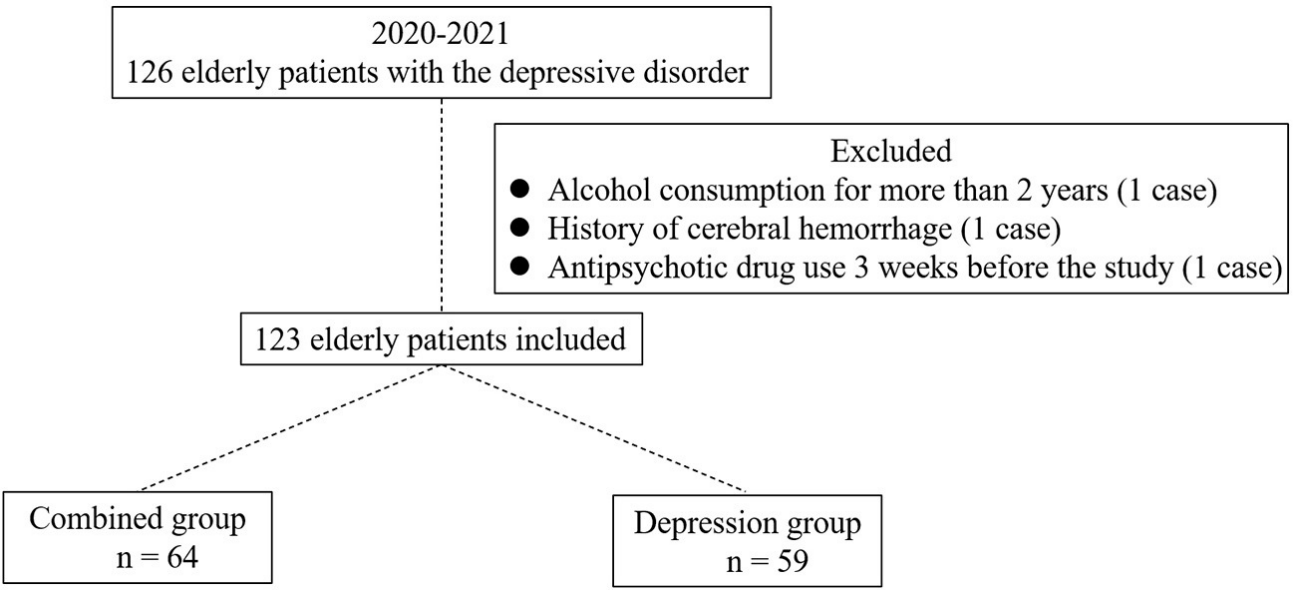
Enrollment of elderly patients with depressive disorder. A total of 126 elderly patients with depressive disorder were primarily enrolled. One case was excluded for alcohol consumption for more than 2 years, one case was excluded for history of cerebral hemorrhage, and one case was excluded for antipsychotic drug use 3 weeks before the study. A total of 123 patients were finally included, among which 64 patients were divided into the combined group, and the others were divided into the depression group.

A total of 123 elderly patients, including 64 patients in the comorbid group and 59 patients in the depressive disorder group, and 70 healthy controls, were retrospectively included in this study (Figure 1). The incidence of depressive disorder combined with cognitive impairment was 52.03% (64/123). There were no significant differences in gender, age, marital status, education background, and smoking among the three groups (*P* > 0.05). The incidence of moderate to severe cognitive impairment in the combined group was significantly higher than that in the depressive group (*P* = 0.003; Table 1).

**Table 1:** Comparison of general conditions between the two groups.

**Item**	**Combined group**	**Depressive disorder**	**Healthy group**	**Statistics**	***P***
	**(*n* = 64)**	**group (*n* = 59)**	**(*n* = 70)**	**(Z/F)**	
Gender (*n*, %)				0.022	0.989
Male	30 (46.88)	27 (45.76)	32 (45.71)		
Female	34 (53.13)	32 (54.23)	38 (54.29)		
Age (years)	72.19 ± 8.76	72.21 ± 9.01	72.28 ± 9.12		
Marital status (*n*, %)				2.546	0.636
Widowed	13 (20.31)	10 (16.69)	15 (21.43)		
Married	49 (76.56)	48 (81.36)	55 (78.57)		
Never married	2 (3.28)	1 (1.69)	0 ()		
Education level (*n*, %)				3.728	0.881
Junior high and below	50 (78.13)	46 (77.97)	55 (78.57)		
High school or equivalent	8 (12.50)	7 (11.86)	10 (14.29)		
Junior college student	4 (6.25)	3 (5.08)	5 (7.14)		
Undergraduate	2 (3.13)	3 (5.08)	5 (7.14)		
Family history of depression (*n*, %)	9 (14.06)	6 (10.17)	4 (5.71)		
Inducement (*n*, %)					
Physical disease	47 (73.44)	43 (22.03)	–	3.485	0.062
Anxiety symptom	40 (62.50)	37 (62.71)	–	2.164	0.141
Life events	39 (60.94)	37 (62.71)	–	0.144	0.705
Medication					
Antianxietic	47 (73.44)	42 (71.19)		0.078	0.782
Benzodiazepine	33 (51.56)	29 (49.15)		0.071	0.789
Antipsychotic drug	17 (26.56)	14 (23.73)		0.851	0.359
Single antidepressant	34 (53.13)	39 (66.10)		2.143	0.143
Combined with antidepressants	16 (25.00)	14 (23.73)		1.433	0.231
Non-benzodiazepine designer	2 (3.13)	3 (5.08)		0.302	0.582
Complication of chronic disease (*n*, %)					
1–2 types	29 (45.31)	26 (44.07)		0.004	0.951
3–6 types	30 (46.88)	28 (47.46)		0.739	0.392
≥7 types	4 (6.25)	3 (5.08)		0.078	0.779
Severity of depression (*n*)				4.715	0.003
Mild	42 (65.63)	26 (44.07)			
Moderate to severe	33 (51.56)	19 (32.20)			

### Comparison of clinical symptoms of depression between the combined group and the depressive disorder group

The incidence of somatic symptoms, suicidal tendency, retardation of thinking, diminution of energy, anxiety, and sleep disorder in the combined group was significantly higher than that in the depressive disorder group, and the difference was statistically significant (*P* < 0.05; Table 2).

**Table 2:** Comparison of clinical symptoms between the combined group and the depressive disorder group.

**Item**	**Combined**	**Depressive**	**χ^2^**	***P***
	**group**	**disorder**	
	**(*n* = 64)**	**group (*n* = 59)**	
Somatization symptom (*n*, %)	30 (46.88)	16 (27.12)	5.118	0.024
Mood change (*n*, %)	38 (59.38)	17 (28.81)	11.599	0.001
Suicidal tendency (*n*, %)	12 (18.75)	3 (5.08)	5.354	0.021
Retardation of thinking (*n*, %)	33 (51.56)	14 (23.73)	10.073	0.002
Diminution of energy (*n*, %)	37 (57.81)	23 (38.98)	4.754	0.029
Anxiety (*n*, %)	42 (65.63)	25 (42.37)	6.693	0.011
Sleep disorder (*n*, %)	50 (78.13)	42 (71.19)	4.636	0.031

### Clinical symptoms of depression and its relationship with cognitive impairment in patients with depressive disorder

Spearman rank correlation analysis suggested that somatic symptom, mood change, suicidal tendency, retardation of thinking, diminution of energy, anxiety, and sleep disorder were negatively correlated with cognitive impairment, respectively (*r* = −0.161, −0.672, −0.262, −0.871, −0.421, −0.571, −0.512, *P* < 0.001).

### Logistic regression was used to evaluate the influencing factors of cognitive function in patients with depressive disorder

Logistic regression analysis indicated that the influencing factors of cognitive dysfunction in patients with depressive disorder were somatic symptom [odds ratio (OR) = 0.532, 95% confidence interval (CI): 0.298–0.893, *P* = 0.013], suicidal ideation (OR = 0.927, 95% CI: 0.701–1.212, *P* < 0.001), retardation of thinking (OR = 2.217, 95% CI: 0.053–0.786, *P* < 0.001), diminution of energy (OR = 0.111, 95% CI: 0.041–0.231, *P* < 0.001), anxiety (OR = 0.072, 95% CI: 0.029–0.254, *P* < 0.001), and sleep disorder (OR = 0.871, 95% CI: 0.783–0.973, *P* = 0.014; Table 3).

**Table 3:** Logistic regression analysis of influencing factors of cognitive function in patients with depressive disorder.

**Item**	**β**	**SE**	**Wald**	**OR (95% CI)**	***P***
Somatic symptom	−0.607	0.248	5.231	0.532 (0.298–0.893)	0.013
Mood change	−0.121	0.053	4.631	0.871 (0.778–0.982)	<0.001
Suicidal tendency	−0.069	0.012	16.371	0.927 (0.701–1.212)	<0.001
Retardation of thinking	−1.456	0.632	5.187	2.217 (0.053–0.786)	<0.001
Diminution of energy	−2.176	0.418	21.298	0.111 (0.041–0.231)	<0.001
Anxiety	−2.498	0.621	15.287	0.072 (0.029–0.254)	<0.001
Sleep disorder	−0.127	0.051	5.761	0.871 (0.783–0.973)	0.014

OR, odds ratio; CI, confidence interval.

## Discussion

Depressive disorder is a chronic mental disease, with significant and persistent low mood as the main clinical symptoms. It was reported that the incidence of depressive disorder in China was increasing year by year (10). In elderly patients, depressive disorder is not only accompanied by physical symptoms but also interwoven with depressive mood. This state has a huge negative impact on patients' physical and mental health and quality of life (10). Cognitive function is the function of the brain to perform higher-level activities including thinking, imagination, memory, and executive functions (11). Cognitive impairment could affect patients' quality of life in the long term. Elderly patients with depression disorder with cognitive dysfunction, visual-spatial ability, executive function, information processing speed, episodic memory, and other disorders. Therefore, the treatment goal of elderly depressive disorder not only includes the improvement of their depression symptoms, improves cognitive function, and then restores the patient's social function (11). Studies have pointed out that it is of great significance to analyze the relationship between clinical symptoms and cognitive impairment in elderly patients with the depressive disorder for early diagnosis and timely treatment intervention (12).

Depression disorder combined with cognitive dysfunction has a high incidence in the elderly population. Depression is associated with increased risk for cognitive function (13). Bellou et al. (14) reported that up to 60% of patients with the depressive disorder also met the diagnosis of cognitive dysfunction. In this study, the incidence of depressive disorder with cognitive impairment was 52.03% (64/123), indicating a high incidence of depressive disorder with cognitive impairment. The incidence of somatic diseases in the elderly is significantly higher than that in the young and middle-aged, which may be due to the increase in age, the incidence of somatic disease-induced depression also increases (15). Studies (16) have shown that the cognitive impairment of patients with the depressive disorder was mainly related to the impairment of the frontal-parietal lobe cognitive control network. Of these, executive function, memory, and attention were reported to be the most commonly affected areas. Previous research reported that cognitive impairment was a secondary symptom caused by depression, mental retardation, and other depressive disorders (17, 18). The study of Liu et al. (19) suggested that attention and delayed recall were negatively correlated with clinical symptoms in elderly patients with amnestic mild cognitive impairment. This current study also suggested that the clinical symptoms of patients with the depressive disorder were negatively correlated with cognitive impairment, suggesting that the more severe the depression disorder was in the elderly, the worse the cognitive function would be.

Cognitive dysfunction in patients with depressive disorder is affected by many factors. Studies have shown that sleep factors play an important role in the formation and development of cognitive impairment (20). Lack of sleep time is easy to leads to words can only appear as directional power disorder, if it is easy to wake up at night, nightmares and other sleep disturbances, will affect the patient's memory, language expression ability. Aging is associated with changes in sleep structure, decreased sleep efficiency, and increased risk of cognitive impairment. Anxiety is a common symptom in people with depressive disorders. Fang (21) reported that the somatic symptoms of elderly patients with a depressive disorder are mainly anxiety and suicidal consciousness. Brain imaging studies indicated that anxiety symptoms in patients with depressive disorders were associated with abnormal activity of local neurons in brain regions (22). Liu et al. (23) found that depression and anxiety were independent risk factors for cognitive dysfunction. The results of this study suggested that the influencing factors of cognitive function in patients with depressive disorder were somatization symptoms, suicidal ideation, thought retardation, energy loss, anxiety, and sleep disorder. At the same time, cognitive dysfunction had an impact on depressive disorders. Peng et al. (24) pointed out that cognitive dysfunction can aggravate a depressive mood. Li et al. (25) pointed out that cognitive function has an impact on suicidal ideation in depressed patients. According to clinical experience, the author thinks that depression disorder combined with cognitive dysfunction can be prevented from the following aspects: (1) Actively adopting symptomatic drug treatment and timely adjusting medication regimen according to the specific situation of patients to reduce the impact of depression on sleep disorders and cognitive impairment; (2) Strengthen health education. Combined with the education level of patients, the health knowledge education was strengthened in the form of pictures and interesting videos, and the influencing factors of depression disorder, sleep disorder, and cognitive impairment were informed to patients as well as daily self-management methods; (3) We should pay more attention to the elderly and education, comprehensively evaluate the individual's actual situation, and adopt memory games to ensure the continuous progress of cognitive impairment; (4) Targeted psychological intervention, guide patients to take a walk, listen to music, do manual work, embroidery and other ways to divert attention, relieve their depression, reduce the severity of the primary symptoms; (5) Patients should be informed to make a sleep schedule based on their actual situation, take regular rest in strict accordance with the schedule, reduce the noise of the sleep environment, and keep the sleep environment quiet. During the day or 30 min before falling asleep at night, they can properly take a walk, do exercises, and other sports to keep the balance of rest and movement.

This study also had the following limitations: The first is the limited sample size of the study Secondly, the cases included in this study were from a single-center, so there may be some bias in the selection of patients. Thirdly, the follow-up period is relatively short. Therefore, a more scientific sample size and more comprehensive design are still needed to improve the quality of research results.

## Conclusion

The clinical symptoms of depressive disorder were negatively correlated with cognitive impairment. Somatic symptoms, suicidal tendency, retardation of thinking, diminution of energy, anxiety, and sleep disorder were the risk factors for cognitive impairment.

## Data availability statement

The original contributions presented in the study are included in the article/supplementary material, further inquiries can be directed to the corresponding author/s.

## Ethics statement

The studies involving human participants were reviewed and approved by the First Hospital of Hebei Medical University. The patients/participants provided their written informed consent to participate in this study.

## Author contributions

ZW and BX contributed to the conception and design of the study. GS and WL performed the experiments, collected, and analyzed data. LL, ZZ, ZW, and BX wrote the manuscript. GS, ZW, and BX revised the manuscript. All authors reviewed and approved the final version of the manuscript.

## Conflict of interest

The authors declare that the research was conducted in the absence of any commercial or financial relationships that could be construed as a potential conflict of interest.

## Publisher's note

All claims expressed in this article are solely those of the authors and do not necessarily represent those of their affiliated organizations, or those of the publisher, the editors and the reviewers. Any product that may be evaluated in this article, or claim that may be made by its manufacturer, is not guaranteed or endorsed by the publisher.

## Abbreviations

GDS: Geriatric Depression Scale
ICD-10: International Classification of Diseases
LSD: least significant difference
MoCA: Montreal Cognitive Assessment Scale
SD: standard deviation.
